# Learning Geometry Information of Target for Visual Object Tracking with Siamese Networks

**DOI:** 10.3390/s21237790

**Published:** 2021-11-23

**Authors:** Hang Chen, Weiguo Zhang, Danghui Yan

**Affiliations:** Automation College, Northwestern Polytechnical University, Xi’an 710072, China; zhangwg@nwpu.edu.cn (W.Z.); yandh@mail.nwpu.edu.cn (D.Y.)

**Keywords:** visual object tracking, deformable convolution, deformable cross-correlation, Siamese network

## Abstract

Recently, Siamese architecture has been widely used in the field of visual tracking, and has achieved great success. Most Siamese network based trackers aggregate the target information of two branches by cross-correlation. However, since the location of the sampling points in the search feature area is pre-fixed in cross-correlation operation, these trackers suffer from either background noise influence or missing foreground information. Moreover, the cross-correlation between the template and the search area neglects the geometry information of the target. In this paper, we propose a Siamese deformable cross-correlation network to model the geometric structure of target and improve the performance of visual tracking. We propose to learn an offset field end-to-end in cross-correlation. With the guidance of the offset field, the sampling in the search image area can adapt to the deformation of the target, and realize the modeling of the geometric structure of the target. We further propose an online classification sub-network to model the variation of target appearance and enhance the robustness of the tracker. Extensive experiments are conducted on four challenging benchmarks, including OTB2015, VOT2018, VOT2019 and UAV123. The results demonstrate that our tracker achieves state-of-the-art performance.

## 1. Introduction

Visual object tracking is a very challenging and fundamental problem in the field of computer vision. It aims to track a given object in all frames of video sequence, and give its position and scale through a bounding box. The object being tracked is usually given in the first frame of the video. Different from the detection task, the tracked object is class-agnostic. Visual tracking has a wide range of applications in practical scenes, such as video surveillance [1], human-computer interaction [2] and automatic driving [3]. Although great progress has been made in this field in recent years, it is still a great challenge to construct a high-performance tracker due to the complexity of the tracking scenarios, like occlusion, similar distractors, scale variation and deformation.

Recently, trackers based on Siamese network have become the mainstream. SiamFC [4] is the first method to introduce Siamese network into tracking. It extracts image features of two branches through convolution neural network (CNN) with shared parameters, and then sends image feature pair into cross-correlation module to compute similarity maps. This architecture is very simple and effective, and Siamese trackers achieve good performance in tracking speed and accuracy. The main components of Siamese trackers are feature extraction network and information embedding module. Many subsequent improvements are mostly based on these two aspects. SiamRPN [5] and siamFC++ [6] improve the estimation accuracy of bounding box by introducing regression branch into the information embedding module. SiamRPN++ [7] and SiamDW [8] use deeper convolution network as feature extraction network to enhance the capacity of feature representation.

**Figure 1:**
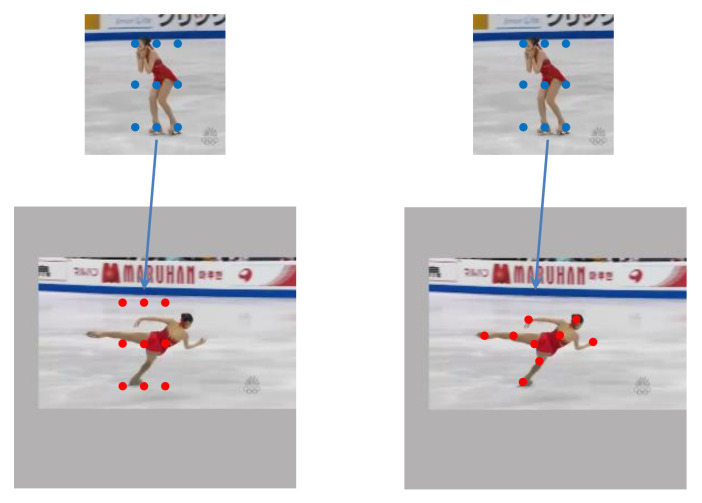
Illustration of traditional cross-correlation and deformable cross-correlation. The left pair of images is the traditional cross-correlation operation, and the right pair is the deformable cross-correlation operation. The blue dots represent the convolution kernel constructed by the template features, and the red dots represent the sampling points of convolution operation on the feature maps of search image.

Although these trackers have achieved great success, there are still some problems. Firstly, the template branch is fixed, which makes the tracker cannot deal with the drastic changes in appearance and long sequence. Secondly, most current Siamese trackers ignore the variation of object geometry in the tracking process, which will reduce the robustness of the tracker, especially when facing deformation, scale variation and rotation. As illustrated in Figure 1, the traditional cross-correlation is limited to a fixed area and cannot adapt to the deformation of the target, which is easy to cause the loss of target information or background interference. From the above considerations, we can see that modeling the appearance changes and object geometry is very important for constructing an efficient tracker.

In order to tackle the above problems, we propose a new information embedding module called deformable cross-correlation for visual object tracking. The idea of deformable cross-correlation comes from deformable convolution network. We model the geometric transformation information by learning a sampling position offset. Compared with the traditional cross-correlation, deformable cross-correlation learns an offset field end-to-end in the information embedding module, which can adapt to the variation of object scale, posture and deformation, reduce the influence of background and ensure the integrity of target features. In addition, to solve the problem of drastic changes of object appearance, we also train an online classification branch to improve the robustness of the tracker. The online classifier includes two modules: attention module and filter module. We send the search image features into the attention module, and use the attention mechanism to suppress the interference information and enhance the target information. In the filter module, a deformable convolution layer is also used to model the object geometry, and the filter is updated in the tracking process to deal with the change of object appearance. The main contributions of this paper are summarized as follows:

- We propose a deformable cross-correlation module to aggregate template information and search information. The geometric information can make the similarity matching process more accurate and improve the tracking performance. In addition, we use attention mechanism and deformable convolution layer to construct an online classifier. Online classification ensures that the tracker can receive the latest information of the target in real time, and improves the ability of the tracker to deal with the changes of the target appearance.
- Using deformable cross-correlation module and online classifier, we construct a target geometry-aware tracker for visual target tracking. This framework is simple and effective. It can adapt to scale variation and deformation in the tracking process, and improve the robustness of the tracker in the tracking process by learning new target information through an online classifier.
- We have conducted extensive experiments on several challenging benchmarks. Experiments show that our proposed tracker outperforms many state-of-the-art trackers.

This paper is organized into five sections, and the remaining content is as follows. The related works are reviewed in Section 2 and the proposed method is described in Section 3. Section 4 presents the experimental results. We conclude this paper in the last section.

## 2. Related Work

In recent years, visual tracking has received increasing attention in computer vision. Deep learning methods have also been introduced into visual tracking, such as FCN [9], DrsNet [10]. In this section, we will review the Siamese trackers and deformable convolution.

### 2.1. Siamese Network Based Visual Tracking

SiamFC [4] and SINT [11] are the first trackers to apply the Siamese network to the tracking field. Siamese structure is very simple and effective. It includes two branches: template branch and search branch. Tracking is achieved by matching template features with each position in search features. In order to estimate the bounding box of the object more accurately, SiamRPN [5] introduces a region proposal networks (RPN) to regress the boxes. SiamRPN++ [7] and SiamDW [8] use deeper convolution network to extract features, which makes full use of the potential of deep networks and improves the representation capacity of features. SiamMask [12] combines instance segmentation and visual tracking to achieve more fine-grained bounding box estimation. According to the idea of multi-stage aggregation in the detection field, C-RPN [13] proposes a cascade tracking framework to continuously refine the tracking results and improve the tracking performance. Although RPN can improve the accuracy of box estimation, it also has some defects: it introduces extra parameters and requires prior knowledge to preset anchors. Recently, many anchor-free based trackers have emerged to solve this problem, like SiamCAR [14], SiamFC++ [6], SiamBAN [15] and Ocean [16]. These methods have achieved the state-of-the-art tracking performance. In addition, attention mechanism is also used in tracking field to improve the robustness and accuracy of tracking. RASNet [17] introduces a variety of attention mechanisms, including off-line trained general attention, residual attention and channel attention. Unlike the trackers above, SiamGAT [18] improves the cross-correlation. It uses graph attention to construct local to local consistency of template features and search features, which makes up for the defect that cross-correlation operation only focuses on global matching and ignores target structure and local information. Most Siamese trackers still have a defect, that is, the template branch is fixed in the whole tracking process, and there is no parameter update, which leads to a certain decline in the stability of the tracker. CFNet [19] and DCFNet [20] integrate the discriminant correlation filter (DCF) into the Siamese framework; that is, DCF is used as a layer in the convolution network, so as to realize online update. Different from updating filter layer, CFCF [21] fine-tunes the feature extraction network through end-to-end training, so as to improve the tracking performance of the tracker.

### 2.2. Deformable Convolutional Networks

In most computer vision tasks, we often face the challenge of geometric change or geometric transformation in scale, attitude, perspective and deformation. The traditional convolution networks inherently cannot model the internal geometric structure of the object, and can only rely on the enhancement of the existing training samples to reduce the impact of geometric structure variation, but still cannot deal with the unknown geometric transformation in the new tasks. Dai et al. [22] propose deformable convolutional networks (DCN) to model geometric transformation, which introduces a 2D offset to the grid sampling position of standard convolution, so the sampling position is not limited by the grid sampling, and the input features can be adaptive to the deformation of the object. Zhu et al. [23] have further proposed an improved DCN, which uses more deformable convolution layers in the practical tasks, and increases the position weight for each sampling position, so that DCNv2 has the ability to focus on the region of interest of the image. In object detection, Yang et al. [24] propose RepPoints, which represents the object more finely as a set of sample points and avoids using rough bounding box to represent the object. In addition, estimated box is obtained directly through sample points, which is easy to use to achieve end-to-end training. Then, Yang et al. [25] propose an improved dense RepPoints, which mainly expands the set of sample points to make the representation of objects more fine-grained and can be applied to segmentation tasks. Some efficient calculation strategies are also proposed to accelerate the calculation of large sample set. Ma et al. [26] introduce the idea of sample point set representation into the field of tracking, and propose RPT to indicate the position of semantic and geometric significance on the target object through training point set. Aiming at appearance variations and dynamic environment, Walia et al. [27] propose to make the tracker adaptive to the appearance of objects through unified graph fusion of multicue.

## 3. Proposed Method

**Figure 2:**
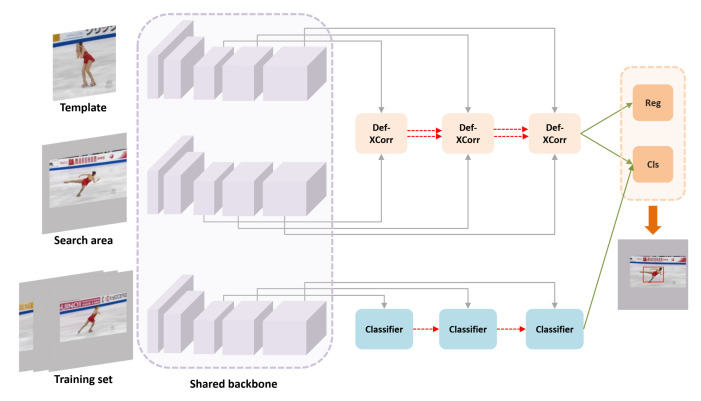
An overview of the proposed target geometric-aware visual tracking framework. The upper part is an off-line trained target estimation sub network, which mainly uses deformable cross-correlation to model the geometric transformation information of the tracking object. Reg represents the regression branch in the classification regression module, and Cls represents the classification branch. The lower part is the online classification sub network. It uses online trained classifiers to deal with the drastic changes of object appearance, supplements the classification results of target estimation sub network, and improves the robustness of the tracker.

As shown in Figure 2, Siamese deformable cross-correlation framework mainly includes four components: shared feature extraction networks, deformable cross-correlation module, classification and regression head and online classification branch. We use the modified ResNet50 as the backbone to extract template features and search features, send the features to the information embedding module for information fusion, and then send the fused information to the classification regression module to obtain the estimation results. In the online classification branch, we use the attention mechanism to enhance the target information, suppress the background information, and construct the filter through the deformable convolution layer. Finally, the results of the two branches are fused to obtain the final tracking results.

### 3.1. Deformable Convolution

**Figure 3:**
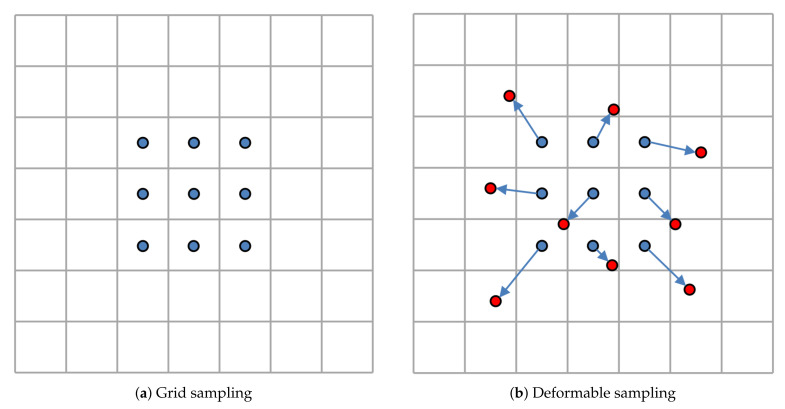
Illustration of 3×3 standard convolution and deformable convolution. (**a**) The grid sampling strategy of standard convolution operation (blue dots). (**b**) In deformable convolution sampling, new sampling positions are obtained by applying offset to the standard sampling positions. The red dots are the new sampling positions, and the blue arrows indicate the offsets.

For notation clarity, we describe the convolution operation in 2D instead of 3D. 2D convolution operation mainly includes two steps: sampling and weighted summation. As illustrated in the Figure 3, the sampling strategy used in traditional convolution operation is grid sampling, and the convolution kernel is used as the weight. The deformable convolution focuses on improving the sampling step, so that the sampling position can be adaptive to the geometric transformation of the object. For a 3×3 kernel with dilation 1, the sampling position of standard convolution can be expressed as follows:

(1) $$S=\left\{\left(-1,-1\right),\left(-1,0\right),...,\left(0,1\right),\left(1,1\right)\right\}.$$

Then, the value of each point *p* on the output feature map *y* can be computed by the following formula,

(2) $$y\left(p\right)=\sum_{s_{i}\in S}w\left(s_{i}\right)\cdot x\left(p+s_{i}\right),$$

where $s_{i}$ indicates position in *S*, *w* is kernel.

For deformable convolution operation, we apply an offset to the position of each sampling point, which relieves the limitation that the sampling point position of convolution operation is fixed, so that the sampling position of deformable convolution operation can be adaptive to the internal structure of the object. Therefore, we have

(3) $$y_{D}\left(p\right)=\sum_{s_{i}\in S}w\left(s_{i}\right)\cdot x\left(p+s_{i}+\Delta s_{i}\right),$$

where $\Delta s_{i}$ is the offset corresponding to position $s_{i}$. Since the offset position $p+s_{i}+\Delta s_{i}$ may not be an integer, the value of sampling point at fractional position can be obtained by linear interpolation.

### 3.2. Siamese Deformable Cross-Correlation

Given two images: template *Z* and search *X*, the traditional Siamese tracker extracts template features and search features, respectively, through the shared backbone φ, and then aggregates the target information through the information embedding function.

(4) $$f\left(Z,X\right)=\varphi_{\theta }\left(Z\right)★\varphi_{\theta }\left(X\right),$$

where θ indicates parameters of backbone, ★ indicates cross-correlation.

Information embedding functions can be divided into cross-correlation and depth-wise cross-correlation. SiamFC uses cross-correlation layer to compute a single channel response map, which takes the whole template feature as a convolution kernel. SiamRPN++ uses depth-wise cross-correlation instead of cross-correlation, which can reduce the computation cost and memory usage. It combines the template feature and search feature of the same channel into pairs, and performs correlation on each channel, respectively. The dimension of the response maps is the same as that of the template features and search features. In this paper, we use depth-wise deformable cross-correlation to aggregate two image information. For the convenience of analysis, we only consider the calculation of a single channel.

**Figure 4:**
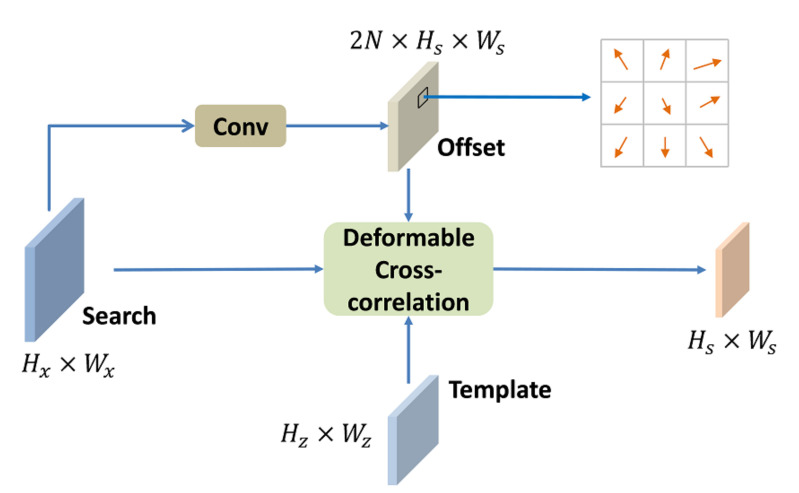
Illustration of deformable cross-correlation. Hx, Wx, Hz, Wz represent the height and width of the search image feature and the template feature, respectively. N represents the number of elements in the template feature ($H_{z}*W_{z}$).

Inspired by DCN, we design a deformable cross-correlation to compute correlation maps. The deformable cross-correlation structure is illustrated in Figure 4. The sizes of search feature and template feature are $H_{x}\times W_{x}$ and $H_{z}\times W_{z}$. The offsets are computed by applying a convolution layer over the search feature. It is worth noting that the spatial resolution of the convolution kernel is consistent with the template feature $H_{z}\times W_{z}$, and the number of convolution kernel is the same as the number of template feature elements *N*. Thus, the size of the offset field output by the convolution layer is $2N\times H_{s}\times W_{s}$, where 2N represents *N* 2-D sampling points. The offset field has the same spatial resolution as the correlation maps. Each point in the offset field indicates a set of sampling offsets for correlation. An example of sampling offset of a 3×3 convolution kernel is given in Figure 4. In the training process, the offset field is learned end-to-end.

We add the offset field information to the cross-correlation calculation process to obtain the deformable cross-correlation operation.

(5) $$f_{D}\left(Z,X\right)=\varphi_{\theta }\left(Z\right){★}_{D}\varphi_{\theta }\left(X\right),$$

where ${★}_{D}$ indicates deformable cross-correlation.

### 3.3. Classification and Regression Module

**Figure 5:**
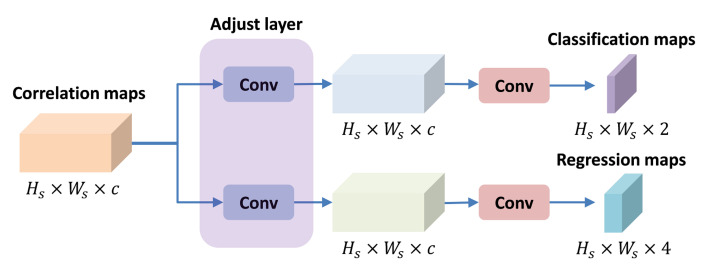
The classification and regression module used in this paper includes two branches: classification branch and regression branch. The adjust layers are convolution layers with kernel size of 1×1.

As shown in Figure 5, after obtaining the correlation maps, we use the classification and regression module to accurately locate the target object. There are some problems in RPN networks, which can only be trained on positive samples (IOU>0.6), and requires prior knowledge to define anchors. All of these limit the performance of RPN networks. On the contrary, the anchor free method can be trained on the samples with small overlap. It does not need predefined anchors and reduces additional parameters. Similar to FCOS [28], this paper uses the idea of center point to construct classification and regression module.

First, we define the regression target. Let the position of point $P_{cls}$ on the classification feature maps be $\left(x_{cls},y_{cls}\right)$, which corresponds to the position of point $P_{in}$ on the input image be $\left(x_{in},y_{in}\right)=\left(\left\lfloor \frac{s}{2}\right\rfloor +x_{cls}s,\left\lfloor \frac{s}{2}\right\rfloor +y_{cls}s\right)$, where *s* is the total stride of the backbone. If $P_{in}$ falls into the groundtruth box, it is regarded as a positive sample, otherwise it is regarded as a negative sample. For regression task, we only consider positive samples. We take the distance between $P_{in}$ and the four sides of the ground truth box as the regression target T=(l,t,r,b), where $l=x_{in}-x_{0}$, $t=y_{in}-y_{0}$, $r=x_{1}-x_{in}$, $b=y_{1}-y_{in}$. $\left(x_{0},y_{0}\right)$ and $\left(x_{1},y_{1}\right)$ represent the upper left corner and the lower right corner of the ground truth box, respectively. The regression branch loss function is

(6) $$L_{reg}=\frac{1}{N_{pos}}\sum_{\left(x_{in},y_{in}\right)\in P_{pos}}L_{IOU}\left(O_{\left(x_{in},y_{in}\right)},T_{\left(x_{in},y_{in}\right)}\right),$$

where $N_{pos}$ indicates the number of positive samples, $P_{pos}$ is the positive sample set, $O_{*}$ is the corresponding predicted bounding box, $L_{IOU}$ is the IOU loss [29].

Different from FCOS, we do not use the center-ness branch, but use a position weighted classification loss.

(7) $$L_{cls}=W_{dist}L_{f}\left(P_{cls},C_{cls}\right),$$

where $C_{cls}$ is classification label for sample $P_{cls}$, $L_{f}$ denotes focal loss [30], $W_{dist}$ is a Gaussian weight function.

Finally, the objective loss is

(8) $$L_{all}=L_{cls}+\alpha L_{reg},$$

where α is used to balance the two parts.

### 3.4. Online Classification Sub-Networks

**Figure 6:**
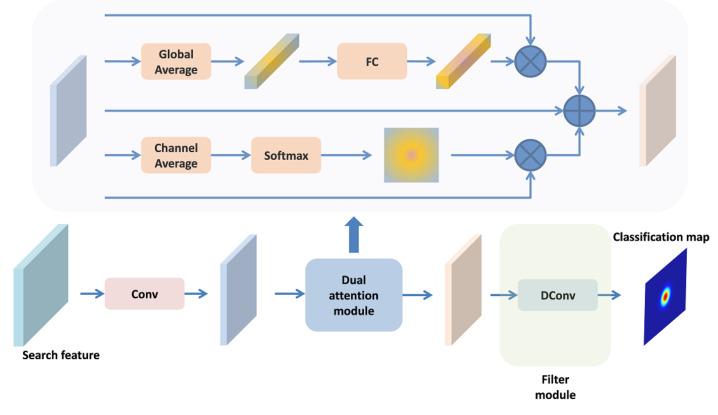
Architecture of online classification sub-network. DConv represents deformable convolution and Conv represents standard convolution. FC represents two-layer fully connected networks.

In the previous subsection, the template feature is fixed, which limits the discriminability of the method. Thus, we propose an online trained classifier to solve this problem, which provides a supplement to the classification branch and improves the robustness and accuracy of the tracker. Similar to ATOM [31] and DROL-RPN [32], we use a lightweight two-layer full convolution networks to construct the classifier, as shown in Figure 6. It is worth noting that the kernel size of the first convolution is 1×1. It is mainly used to reduce the dimension of features and reduce the cost of calculation. The second convolution layer is the deformable convolution layer, which constitutes the filter module we update in the tracking process. In addition, in order to enhance the target feature information and reduce the influence of interference factors, we use a dual attention module to enhance the reduced dimension features.

The attention module consists of two parts: channel attention and position attention. For channel attention, we first use the global average pooling operation to obtain the channel information, and then use a two-layer full connected network to establish the relationship between channels, so as to obtain the channel attention information. For positional attention, we use channel averaging and softmax operation to obtain positional attention information. Finally, channel attention and position attention are fused with the initial features to obtain the final enhanced features.

(9) $$X_{en}=X_{in}+X_{in}⊙C_{a}+X_{in}⊗P_{a},$$

where $C_{a},P_{a}$ represent channel attention and position attention, ⊙ represents channel-wise production, and ⊗ represents point-wise production.

The online training samples come from the previous frames of the current frame. To avoid noise influence such as occlusion, we only choose high-quality frames whose classification score is higher than a certain threshold as the training samples, and the label of the training frame is a binary Gaussian function whose center is located in the center of the target estimation position. In addition, in order to model the geometric information of the object, we introduce the sampling position offset in the deformable convolution layer to construct the deformable classifier. The sampling offset comes from the sampling offsets obtained from the deformable cross-correlation. The filter size in the online classification branch is the same as the template feature size in the deformable cross-correlation.

In the tracking process, the online classifier is initialized in the first frame, and then all parameters except the filter are fixed. This not only reduces the number of update parameters, reduces the amount of calculation, but also maintains the stability of the classifier. In the subsequent online updates, we only update the filter parameters. Similar to ATOM [31], we do not use the stochastic gradient descent (SGD) optimization strategy, but use the conjugate gradient descent method to accelerate the convergence speed.

After calculating the online classification map, we fuse it with the classification results of the matching branch to obtain the final classification map.

(10) $$S_{all}=\beta S_{online}+\left(1-\beta \right)S_{cls},$$

where $S_{online}$ is the output of online classifier, $S_{cls}$ is score map of matching branch, β is parameter to balance two parts.

### 3.5. Multi-Stage Fusion

In many visual tasks, using multi-stage features is an effective strategy. In the field of tracking, such as HCF [33] and C-RPN [13], they all adopt multi-layer features. Shallow features preserve fine texture, shape and other high spatial resolution features, which is conducive to target location. Deep features preserve high robust semantic information, which is used to distinguish objects from the background. The features of different layers are complementary to each other in tracking tasks, and can deal with some challenging scenes, such as motion blur and large deformation.

In this paper, we use ResNet-50 to extract the features, and the output of the last three-stage is used as the feature representation. Similar to SiamRPN++, the last three-stage feature maps have the same spatial resolution, so we can conveniently fuse multi-stage features. For the classification and regression module, we first compute the classification maps and regression maps on all stages, and then fuse all the results by weighted sum.

(11) $$S_{cls}=\sum_{l=3}^{5}\gamma_{l}\cdot S_{cls}^{l},$$

(12) $$R_{all}=\sum_{l=3}^{5}\delta_{l}\cdot R_{l},$$

where $S_{cls}^{l}$ and $R_{l}$ represent classification maps and regression maps of stage *l*. $\gamma_{l}$ and $\delta_{l}$ represent fusion weight for corresponding stage. After obtaining the fusion classification map, it is finally fused with the online classification map to estimate the target.

For the online classification sub network, we use weighted sum to fuse the online classification results,

(13) $$S_{online}=\sum_{l=3}^{5}w_{l}\cdot S_{online}^{l},$$

where $w_{l},l=3,4,5$ are the fusion weights for classification results at all stages. They are trained end-to-end in the network. · represents element-wise production. $S_{online}^{l},l=3,4,5$ are online classification results at all stages.

## 4. Experiments

This section mainly includes four parts: implementation details, comparison with other state-of-the-art (SOTA) trackers, ablation experiments and qualitative comparison.

### 4.1. Implementation Details

We use ResNet-50 which is pre-trained on ImageNet [34] as the backbone to extract the features. Similar to SiamRPN++, we modify the structure of the network to make it suitable for the tracking field. The output of the last three convolution blocks is taken as the multi-stage features. For the fourth and fifth convolution blocks, the down sampling is discarded to preserve more spatial details. Meanwhile, dilated convolutions with different atrous rates are used to improve the receptive field.

The input of off-line training is an image pair, in which the size of template image is 127×127 and the size of search image is 255×255. The image pair is extracted from a video sequence, and the interval is less than 100 frames. Then the object area is cropped according to ground truth, and finally the patch is resized to the required size. The training data sets include Image VID [35], COCO [36], Image DET [35] and YouTube Bounding Box [37].

The whole training process includes 20 epochs, which can be divided into two stages. In the first five epochs, we use warm up learning rate from 0.001 to 0.005 to train the deformable cross-correlation module and classification and regression head, and freeze the backbone parameters. In the last 15 epochs, the learning rate of the whole network decreases exponentially from 0.005 to 0.0005, and the learning rate of the backbone network is set as one tenth of the overall learning rate. Our optimization method is random gradient descent (SGD), in which momentum is set to 0.9 and weight decay is set to 0.0005. The online classification branch uses the high quality frames that have been tracked as the training set. We use Pytorch to implement this method. All experiments are carried out on a PC equipped with NVIDIA Geforce GTX2080TI GPU and Core i7-8700 at 3.2GHZ CPU.

### 4.2. Comparison with Other Sota Trackers

The proposed tracker is compared with other SOTA trackers on several popular benchmarks. These datasets include OTB2015 [38], VOT2018 [39], VOT2019 [40] and UAV123 [41].

**OTB2015.** The OTB2015 dataset is extended from OTB2013 [42], which contains 100 video sequences. It includes many challenging aspects in visual tracking, like Illumination Variation, Scale Variation, and Occlusion and so on. The evaluation is mainly based on two criteria: precision and success rate. The precision measures the distance between the tracking results and the ground truth center, while the success rate measures the overlaps between estimated boxes and ground truth boxes. We compare our tracker with the following SOTA trackers: ECO-HC [43], SiamFC [4], ATOM [31], SiamRPN++ [7], SiamFC++ [6], SiamAtt [44], Ocean [16], SiamR-CNN [45], SiamBAN [15] and DiMP-50 [46].

**Figure 7:**
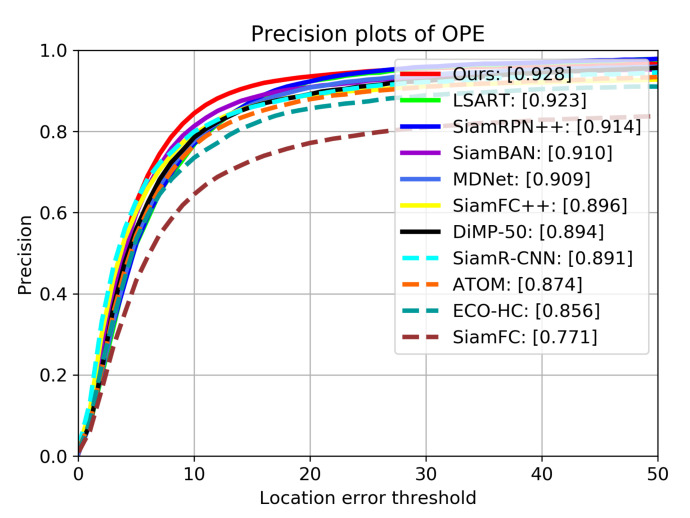
Results of precision on OTB2015. The numbers in the legend indicate the precision scores when the threshold is 20 pixels.

**Figure 8:**
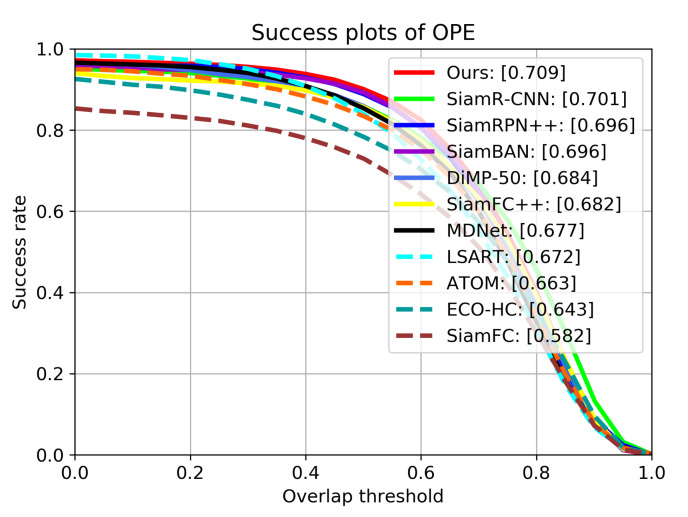
Results of success rate on OTB2015. The numbers in the legend indicate the area under curve (AUC) scores.

Table 1 shows the precision scores, area under curve (AUC) scores and frame per second (FPS) of all trackers on OTB2015, Figure 7 and Figure 8 show the detailed tracking results. Our tracker achieves the best performance of all trackers on both metrics, with AUC score of 0.709 and precision score of 0.928. Compared with SiamRPN++, the performance gains of our tracker on AUC and precision are 1.3% and 1.4%, respectively. This is mainly because our tracker uses deformable cross-correlation and online classification components, so that the tracker can learn more powerful feature representation, so as to improve the accuracy and robustness. Compared with DiMP-50 with online fine-tuning, our tracker also achieves performance gains of 3.4% and 2.3% on precision and success rate.

**VOT2018.** The VOT2018 dataset contains 60 video sequences with different challenges. In contrast to OTB2015, it needs to reinitialize the tracker when tracking fails. The criteria used to evaluate the trackers are accuracy, robustness and expected average overlap (EAO). The accuracy measures the average overlap of the estimation box and ground truth box when the tracking is successful, and the robustness measures the tracking failure rate. The expected average overlap is a combination of the first two criteria, which is used to rank the trackers. We compare our tracker with state-of-the-art trackers, including D3S [47], SiamRPN++ [7], ATOM [31], SiamMask [12], SiamR-CNN [45], DiMP-50 [46], LADCF [39], MFT [39], UPDT [48], SiamFC++ [6].

**Figure 9:**
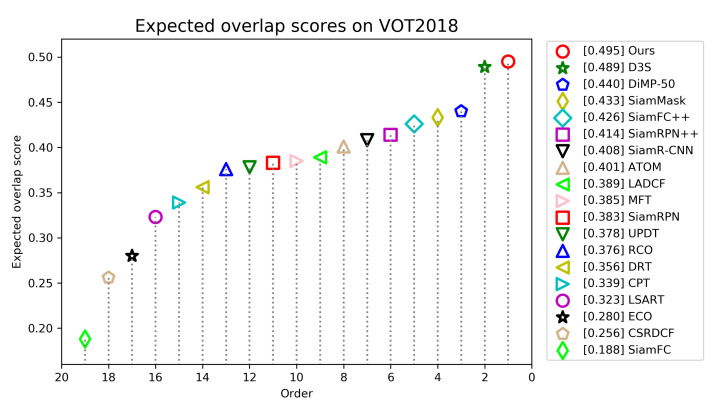
Expected average overlap scores ranking for compared trackers on the VOT2018 benchmark. The trackers on the right have better performance.

Figure 9 shows the EAO scores and rankings of all trackers, and Table 2 gives a detailed comparison of top trackers. Figure 9 shows that our tracker ranks first with the highest EAO score of 0.495. We can see from Table 2 that our tracker achieves the second best score of 0.612 in accuracy and the best score of 0.126 in robust. Compared with LADCF, the best tracker in VOT2018 challenge, our tracker improves the performance of EAO by 10.6%.

**VOT2019.** VOT2019 dataset also contains 60 video sequences, most of which are the same as VOT2018. The difference is that several easiest sequences in VOT2018 is replaced with more challenging sequences. The same measurements as VOT2018 are also exploited for performance evaluation. We compare our track with following trackers: ATOM [31], SiamRPN++ [7], DiMP-50 [46], DRNet [40], ACNT [40], SiamFCOT [40], SiamCRF [40], SiamMask [12], ROAM++ [49], and SPM [50].

**Figure 10:**
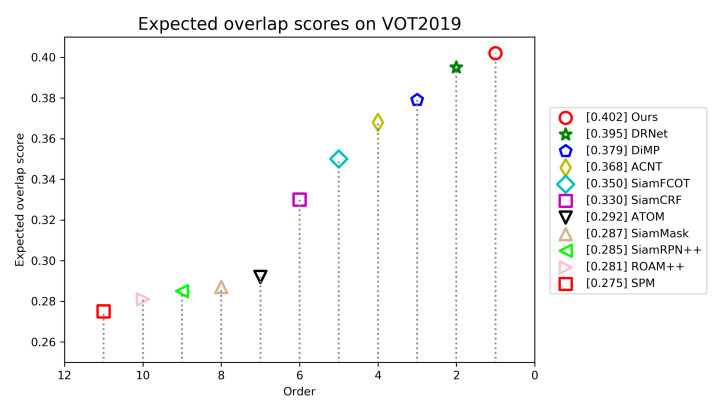
Expected average overlap scores ranking for compared trackers on the VOT2019 benchmark. The trackers on the right have better performance.

As illustrated in Figure 10, our tracker achieves an EAO of 0.402, ranks first and outperforms other trackers. Table 3 shows that our tracker attains the best robustness of 0.220 and third best accuracy of 0.623 among all trackers. ACNT and SiamCRF are slightly superior in terms of accuracy. Compared with DRNet, the best tracker in VOT2019 challenge, our tracker improves the performance of EAO by 0.7%.

**UAV123.** Inherently different from OTB2015 and VOT benchmarks, UAV123 is a dataset captured by Unmanned Aerial Vehicle (UAV), which contains 123 video sequences with an average length of 915 frames. The same measurements as OTB2015 are also exploited for performance evaluation. We compare our tracker with following SOTA trackers: DiMP-50 [46], ATOM [31], ECO [43], SiamRPN++ [7], DaSiamRPN [51], UPDT [48], SiamRPN [5], ECO-HC [43].

Table 4 illustrates the results of the compared trackers. Specifically, our tracker achieves best performance with precision score of 0.872 and AUC score of 0.660. Compared with SiamRPN++ without appearance update and geometric modeling ability, the performance of our tracker in precision and AUC is improved by 6.5% and 4.7%. The Dimp-50 tracker has no geometric structure modeling ability, so its performance is 1.4% and 0.6% lower than our tracker on precision and AUC, respectively.

### 4.3. Ablation Experiment

In this subsection, ablation experiments are performed to illustrate the influence of each components of the tracker proposed in this paper. We conduct ablation experiments on OTB2015 and use SiamFC++ with a backbone of ResNet-50 as the baseline. The components to be analyzed are deformable cross-correlation (Def-XCorr), online classification (OC) and multi-stage aggregation (MSA). As illustrated in Table 5, the improvement gains for three components are 2.3%, 0.4%, 0.7%, respectively.

We can see that Def-XCorr component contributes the most to the performance gain of the tracker. It is proven that the deformable cross-correlation is beneficial to more accurate target estimation. This is partly because it models the geometric structure of the object, makes the information embedding more reasonable, and can adapt to the deformation of the object. OC component introduces new appearance information of the object into the tracker, which can deal with the appearance change factors of the object to a certain extent, so a AUC performance gain of 0.4% is obtained. MSA component enables the tracker to adopt different stages of convolution features, improves the representation ability of the learned object features, and further improves the performance of 0.7% on AUC. At last, the combination of all components makes our tracker achieve the best performance.

### 4.4. Qualitative Comparison

**Figure 11:**
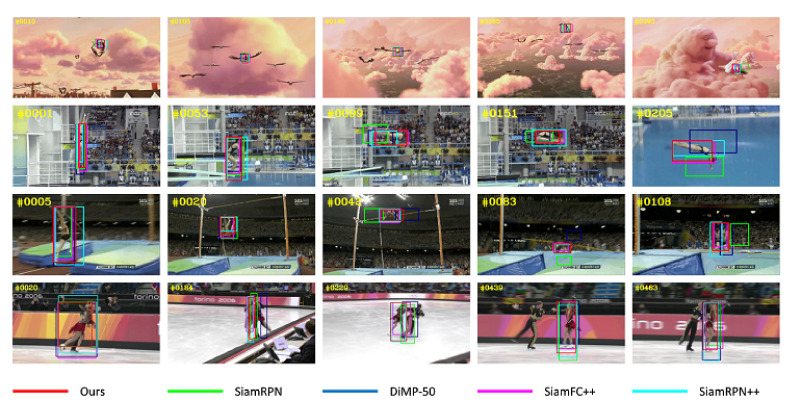
Qualitative comparisons with 4 state-of-the-art trackers.

We compared our tracker with several other advanced trackers quantitatively, and Figure 11 shows the qualitative results on the experimental dataset. The video sequences tested are several challenging scenes selected from OTB2015 benchmark, namely: Bird, Diving, Jump and Skating2-1. We can see that our tracker can model the local information and internal geometry of objects, so it can deal with the deformation and partial occlusion of non-rigid objects. The experimental results show that our tracker achieves the best tracking performance in all test sequences.

**Figure 12:**
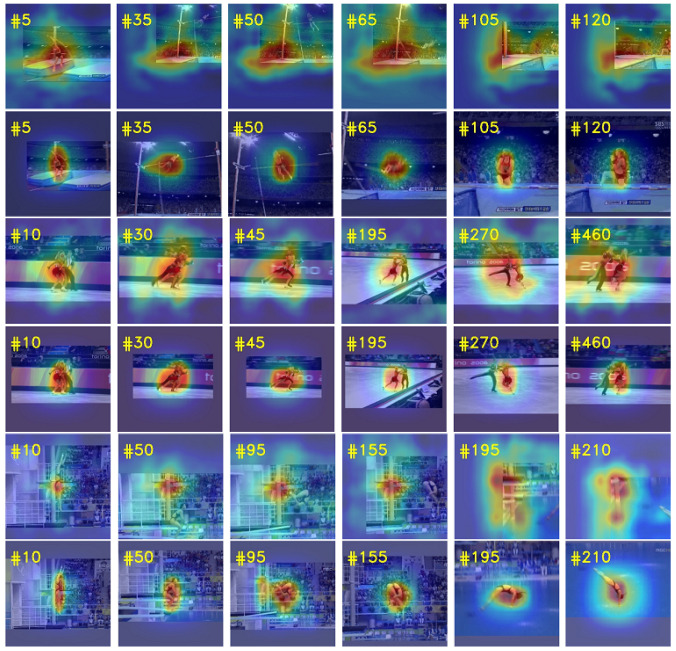

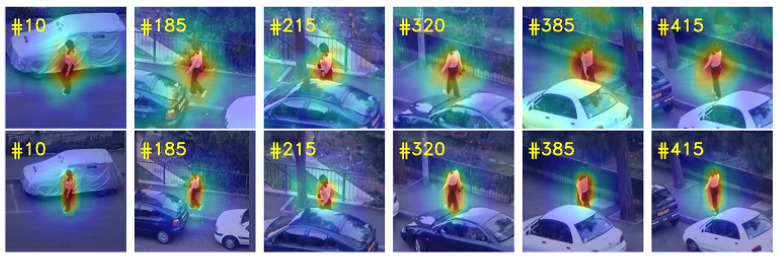
Visualization of response maps. The figure contains four video sequences, and each row represents the tracking results of the corresponding tracker on the sequence. The odd rows represent the response maps of the plain cross-correlation tracker, and the even rows represent the response maps of the deformable cross-correlation tracker.

In order to further illustrate the effectiveness of our proposed method in dealing with the challenges of occlusion and object deformation, we use deformable cross-correlation and plain cross-correlation to build two different trackers, respectively, test them on multiple video sequences with occlusion and deformation factors, and visualize the response of tracking results, as shown in Figure 12. The four video sequences used for the test are Jump, Skating2-1, Diving and Woman from top to bottom. From Figure 12, we can see that the human body in the sequence Jump and Dividing has undergone severe deformation. The plain cross-correlation does not have the ability of local information modeling and internal geometry modeling, resulting in tracking drift and eventually loss of target. In Jump and Dividing sequences, the plain cross-correlation tracker lost the target in frames 35 and 50, respectively, and never recovered. In contrast, the response maps of deformable cross-correlation tracker are very accurate, and the tracker always focuses on the deformed human body. In Skating2-1, there are similar distractor and occlusion factors in video sequences. It can be seen from frames 270 and 460 of Skating2-1 that the deformable cross-correlation tracker has a more accurate response map and is less affected by interferences, while the response map of the plain cross-correlation tracker is more divergent and cannot accurately locate and track the target. In Woman sequence, the woman’s lower body is occluded by a vehicle during walking. From frames 320 to 415 in Woman, it can be seen that in case of partial occlusion, the deformable cross-correlation tracker can quickly focus on the upper body of women, then quickly return to focus on the whole body when passing the vehicle, and can always track the target robustly. When the plain cross-correlation tracker faces partial occlusion, the performance of the tracker decreases and produces strong response in the background. In addition, the response maps of deformable cross-correlation tracker in all sequences are more compact and less disturbed by background information.

## 5. Conclusions

In this work, We propose a geometry-aware tracker to model the geometric structure of the target. Our tracker mainly includes two parts: offline trained target estimation sub network and online classification sub network. We integrate the idea of deformable convolution into these two parts to realize the learning of geometric information. Compared with cross-correlation based trackers, our method can sample the search feature area by learning an offset field, so that it can adapt to the structural variations of the target and achieve more accurate visual tracking. Besides, online classification sub-network is used to deal with the appearance changes of target and provide supplementary information for the tracking process. Comprehensive evaluations on multiple benchmarks indicate that our method can achieve leading performance. In future work, we will explore a backbone network that can model the geometric structure of the object for visual tracking.

## Figures and Tables

**Table 1:** Comparison with the state-of-the-art trackers on OTB2015. The numbers in column of AUC indicate area under curve scores. The numbers in column of Precision are the scores when the threshold is 20 pixels. Red, green and blue represent the first, second and third rank, respectively.

Tracker	AUC	Precision	FPS
ECO-HC	0.643	0.856	60
SiamFC	0.582	0.771	86
ATOM	0.663	0.874	30
SiamRPN++	0.696	0.914	35
SiamFC++	0.682	0.896	90
LSART	0.672	0.923	1
MDNet	0.677	0.909	1
SiamR-CNN	0.701	0.891	4.7
SiamBAN	0.696	0.910	40
DiMP-50	0.684	0.894	43
Ours	0.709	0.928	30

**Table 2:** Detailed experimental results of several top ranked trackers on VOT2018. Red, green and blue represent the first, second and third rank, respectively.

Tracker	EAO ↑	Accuracy ↑	Robust ↓
SiamRPN++	0.414	0.600	0.234
D3S	0.489	0.640	0.150
SiamFC++	0.426	0.587	0.183
ATOM	0.401	0.590	0.204
DiMP-50	0.440	0.597	0.153
LADCF	0.389	0.503	0.159
SiamR-CNN	0.408	0.610	0.220
Ours	0.495	0.628	0.126

**Table 3:** Detailed experimental results of all compared trackers on VOT2019. Red, green and blue represent the first, second and third rank, respectively.

Tracker	EAO ↑	Accuracy ↑	Robust ↓
ATOM	0.292	0.603	0.411
SiamRPN++	0.285	0.599	0.482
DiMP-50	0.379	0.594	0.278
DRNet	0.395	0.605	0.261
ACNT	0.368	0.626	0.278
SiamFCOT	0.350	0.601	0.386
SiamCRF	0.330	0.625	0.296
SiamMask	0.287	0.594	0.461
ROAM++	0.281	0.561	0.438
SPM	0.275	0.577	0.507
Ours	0.402	0.623	0.220

**Table 4:** Results of precision and area under curve (AUC) on dataset UAV123. Red, green and blue represent the first, second and third rank, respectively.

Tracker	Precision	AUC
DiMP-50	0.858	0.654
ATOM	0.856	0.643
ECO	0.741	0.525
SiamRPN++	0.807	0.613
DaSiamRPN	0.795	0.585
UPDT	0.780	0.547
SiamRPN	0.748	0.527
SiamFC	0.648	0.485
Ours	0.872	0.660

**Table 5:** Ablation study of our tracker on OTB2015. Baseline represents a modified SiamFC++ with a replaced backbone of ResNet-50. Def-XCorr, OC, MSA represent deformable cross-correlation, online classification and multi-stage aggregation, respectively.

Baseline	Def-XCorr	OC	MSA	Precision	AUC	ΔAUC
✔				0.898	0.675	-
✔	✔			0.920	0.698	2.3%
✔	✔	✔		0.922	0.702	0.4%
✔	✔	✔	✔	0.928	0.709	0.7%

## Data Availability

Data sharing not applicable.

## References

[1] Emami A., Dadgostar F., Bigdeli A., Lovell B.C. Role of Spatiotemporal Oriented Energy Features for Robust Visual Tracking in Video Surveillance. Proceedings of the 2012 IEEE Ninth International Conference on Advanced Video and Signal-Based Surveillance (AVSS).

[2] Liu L., Xing J., Ai H., Ruan X. Hand posture recognition using finger geometric feature. Proceedings of the 21st International Conference on Pattern Recognition (ICPR).

[3] Lee K., Hwang J. (2015). On-Road Pedestrian Tracking Across Multiple Driving Recorders. IEEE Trans. Multimed..

[4] Bertinetto L., Valmadre J., Henriques J.F., Vedaldi A., Torr P.H.S. Fully-convolutional siamese networks for object tracking. Proceedings of the European Conference on Computer Vision (ECCV Workshops).

[5] Li B., Yan J., Wu W., Zhu Z., Hu X. High Performance Visual Tracking with Siamese Region Proposal Network. Proceedings of the IEEE/CVF Conference on Computer Vision and Pattern Recognition (CVPR).

[6] Xu Y., Wang Z., Li Z., Yuan Y. SiamFC++: Towards Robust and Accurate Visual Tracking with Target Estimation Guidelines. Proceedings of the AAAI Conference on Artificial Intelligence (AAAI).

[7] Li B., Wu W., Wang Q., Zhang F., Xing J., Yan J. SiamRPN++: Evolution of Siamese Visual Tracking With Very Deep Networks. Proceedings of the IEEE/CVF Conference on Computer Vision and Pattern Recognition (CVPR).

[8] Zhang Z., Peng H. Deeper and Wider Siamese Networks for Real-Time Visual Tracking. Proceedings of the IEEE/CVF Conference on Computer Vision and Pattern Recognition (CVPR).

[9] Long J., Shelhamer E., Darrell T. Fully Convolutional Networks for Semantic Segmentation. Proceedings of the IEEE/CVF Conference on Computer Vision and Pattern Recognition (CVPR).

[10] Yu L., Fan G. (2021). DrsNet: Dual-resolution semantic segmentation with rare class-oriented superpixel prior. Multimed. Tools Appl..

[11] Tao R., Gavves E., Smeulders A.W.M. Siamese Instance Search for Tracking. Proceedings of the IEEE Conference on Computer Vision and Pattern Recognition (CVPR).

[12] Wang Q., Zhang L., Bertinetto L., Hu W., Torr P.H.S. Fast Online Object Tracking and Segmentation: A Unifying Approach. Proceedings of the IEEE/CVF Conference on Computer Vision and Pattern Recognition (CVPR).

[13] Fan H., Ling H. Siamese Cascaded Region Proposal Networks for Real-Time Visual Tracking. Proceedings of the IEEE/CVF Conference on Computer Vision and Pattern Recognition (CVPR).

[14] Guo D., Wang J., Cui Y., Wang Z., Chen S. SiamCAR: Siamese Fully Convolutional Classification and Regression for Visual Tracking. Proceedings of the IEEE/CVF Conference on Computer Vision and Pattern Recognition (CVPR).

[15] Chen Z., Zhong B., Li G., Zhang S., Ji R. Siamese Box Adaptive Network for Visual Tracking. Proceedings of the IEEE/CVF Conference on Computer Vision and Pattern Recognition (CVPR).

[16] Zhang Z., Peng H., Fu J., Li B., Hu W. Ocean: Object-Aware Anchor-Free Tracking. Proceedings of the European Conference on Computer Vision (ECCV).

[17] Wang Q., Teng Z., Xing J., Gao J., Hu W., Maybank S. Learning Attentions: Residual Attentional Siamese Network for High Performance Online Visual Tracking. Proceedings of the IEEE/CVF Conference on Computer Vision and Pattern Recognition (CVPR), Salt Lake City.

[18] Guo D., Shao Y., Cui Y., Wang Z., Zhang L., Shen C. (2020). Graph Attention Tracking. arXiv.

[19] Valmadre J., Bertinetto L., Henriques J., Vedaldi A., Torr P.H.S. End-to-End Representation Learning for Correlation Filter Based Tracking. Proceedings of the IEEE/CVF Conference on Computer Vision and Pattern Recognition (CVPR).

[20] Wang Q., Gao J., Xing J., Zhang M., Hu W. (2017). DCFNet: Discriminant Correlation Filters Network for Visual Tracking. arXiv.

[21] Gundogdu E., Alatan A.A. (2018). Good Features to Correlate for Visual Tracking. IEEE Trans. Image Process..

[22] Dai J., Qi H., Xiong Y., Li Y., Zhang G., Hu H., Wei Y. Deformable Convolutional Networks. Proceedings of the IEEE International Conference on Computer Vision (ICCV).

[23] Zhu X., Hu H., Lin S., Dai J. Deformable ConvNets V2: More Deformable, Better Results. Proceedings of the IEEE/CVF Conference on Computer Vision and Pattern Recognition (CVPR).

[24] Yang Z., Liu S., Hu H., Wang L., Lin S. RepPoints: Point Set Representation for Object Detection. Proceedings of the IEEE/CVF International Conference on Computer Vision (ICCV).

[25] Yang Z., Xu Y., Xue H., Zhang Z., Urtasun R., Wang L., Lin S., Hu H. (2020). Dense RepPoints: Representing Visual Objects with Dense Point Sets. arXiv.

[26] Ma Z., Wang L., Zhang H., Lu W., Yin J. RPT: Learning Point Set Representation for Siamese Visual Tracking. Proceedings of the European Conference on Computer Vision (ECCV Workshops).

[27] Walia G.S., Ahuja H., Kumar A., Bansal N., Sharma K. (2020). Unified Graph-Based Multicue Feature Fusion for Robust Visual Tracking. IEEE Trans. Cybern..

[28] Tian Z., Shen C., Chen H., He T. FCOS: Fully Convolutional One-Stage Object Detection. Proceedings of the IEEE/CVF International Conference on Computer Vision (ICCV).

[29] Yu J., Jiang Y., Wang Z., Cao Z., Huang T. UnitBox: An Advanced Object Detection Network. Proceedings of the 24th ACM international conference on Multimedia (ACMMM).

[30] Lin T., Goyal P., Girshick R., He K., Dollár P. (2017). Focal Loss for Dense Object Detection. IEEE Trans. Pattern Anal. Mach. Intell..

[31] Danelljan M., Bhat G., Khan F.S., Felsberg M. ATOM: Accurate Tracking by Overlap Maximization. Proceedings of the IEEE/CVF Conference on Computer Vision and Pattern Recognition (CVPR).

[32] Zhou J., Wang P., Sun H. Discriminative and Robust Online Learning for Siamese Visual Tracking. Proceedings of the AAAI Conference on Artificial Intelligence (AAAI).

[33] Ma C., Huang J., Yang X., Yang M. Hierarchical Convolutional Features for Visual Tracking. Proceedings of the IEEE International Conference on Computer Vision (ICCV).

[34] Deng J., Dong W., Socher R., Li L., Li K., Li F.F. ImageNet: A large-scale hierarchical image database. Proceedings of the IEEE Conference on Computer Vision and Pattern Recognition (CVPR).

[35] Russakovsky O., Deng J., Su H., Krause J., Satheesh S., Ma S., Huang Z., Karpathy A., Khosla A., Bernstein M. (2015). ImageNet Large Scale Visual Recognition Challenge. Int. J. Comput. Vis..

[36] Lin T.Y., Maire M., Belongie S., Hays J., Zitnick C.L. Microsoft COCO: Common Objects in Context. Proceedings of the European Conference on Computer Vision (ECCV).

[37] Real E., Shlens J., Mazzocchi S., Pan X., Vanhoucke V. YouTube-BoundingBoxes: A Large High-Precision Human-Annotated Data Set for Object Detection in Video. Proceedings of the IEEE Conference on Computer Vision and Pattern Recognition (CVPR).

[38] Wu Y., Lim J., Yang M. (2015). Object Tracking Benchmark. IEEE Trans. Pattern Anal. Mach. Intell..

[39] Kristan M., Leonardis A., Matas J., Felsberg M., He Z., Pflugfelder R., Zajc L.C., Vojir T., Bhat G., Lukezic A. The Sixth Visual Object Tracking VOT2018 Challenge Results. Proceedings of the European Conference on Computer Vision Workshops (ECCVW).

[40] Kristan M., Matas J., Leonardis A., Felsberg M., Pflugfelder R., Kamarainen J., Zajc L.C., Drbohlav O., Lukezic A., Berg A. The Seventh Visual Object Tracking VOT2019 Challenge Results. Proceedings of the IEEE/CVF International Conference on Computer Vision Workshops (ICCVW).

[41] Mueller M., Smith N., Ghanem B. A Benchmark and Simulator for UAV Tracking. Proceedings of the European Conference on Computer Vision (ECCV).

[42] Wu Y., Lim J., Yang M. Online Object Tracking: A Benchmark. Proceedings of the IEEE Conference on Computer Vision and Pattern Recognition (CVPR).

[43] Danelljan M., Bhat G., Khan F.S., Felsberg M. ECO: Efficient Convolution Operators for Tracking. Proceedings of the IEEE Conference on Computer Vision and Pattern Recognition (CVPR).

[44] Yang K., He Z., Zhou Z., Fan N. (2020). SiamAtt: Siamese attention network for visual tracking. Knowl.-Based Syst..

[45] Voigtlaender P., Luiten J., Torr P.H.S., Leibe B. Siam R-CNN: Visual Tracking by Re-Detection. Proceedings of the IEEE/CVF Conference on Computer Vision and Pattern Recognition (CVPR).

[46] Bhat G., Danelljan M., Gool L.V., Timofte R. Learning Discriminative Model Prediction for Tracking. Proceedings of the IEEE/CVF International Conference on Computer Vision (ICCV).

[47] Lukežič A., Matas J., Kristan M. D3S—A Discriminative Single Shot Segmentation Tracker. Proceedings of the IEEE/CVF Conference on Computer Vision and Pattern Recognition (CVPR).

[48] Bhat G., Johnander J., Danelljan M., Khan F.S., Felsberg M. Unveiling the Power of Deep Tracking. Proceedings of the European Conference on Computer Vision (ECCV).

[49] Yang T., Xu P., Hu R., Chai H., Chan A.B. ROAM: Recurrently Optimizing Tracking Model. Proceedings of the IEEE/CVF Conference on Computer Vision and Pattern Recognition (CVPR).

[50] Wang G., Luo C., Xiong Z., Zeng W. SPM-Tracker: Series-Parallel Matching for Real-Time Visual Object Tracking. Proceedings of the IEEE/CVF Conference on Computer Vision and Pattern Recognition (CVPR).

[51] Zhu Z., Wang Q., Li B., Wu W., Yan J., Hu W. Distractor-Aware Siamese Networks for Visual Object Tracking. Proceedings of the European Conference on Computer Vision (ECCV).

